# La rétinopathie de Purtscher dans le Lupus

**DOI:** 10.11604/pamj.2015.22.154.6528

**Published:** 2015-10-16

**Authors:** Siham Chariba, Rajae Daoudi

**Affiliations:** 1 Service d'Ophtalmologie, Hôpital des Spécialités, Rabat, Maroc

**Keywords:** Purtscher, lupus, nodules dysoriques, Purtscher, lupus, dysoric nodules

## Image en medicine

Une jeune patiente de 35 ans se présente pour une baisse brutale d'acuité visuelle. L'examen trouve une acuité à « compte les doigts » avec au fond d’œil une papille dysversée avec une hémorragie en flammèche et quelques plages blanchâtres superficielles autour de la papille et en supra fovéolaire. Cet aspect nous évoque la rétinopathie de Purtscher. La pseudorétinopathie de Purtscher peut être retrouvée chez des patients présentant une pancréatite aiguë, une insuffisance rénale, une embolie amniotique ou être secondaire à des injections de corticoïdes ou de produits anesthésiques autour de l'orbite. Elle peut également s'observer dans des maladies immunitaires telles que la dermatomyosite, la sclérodermie ou le lupus. C'est ainsi qu'un bilan complet est réalisé et retrouve un Lupus. La patiente est mise sous bolus de solumédrol relayés par une corticothérapie par voie orale à doses dégressives avec une bonne récupération fonctionnelle au bout de quelques mois.

**Figure 1:**
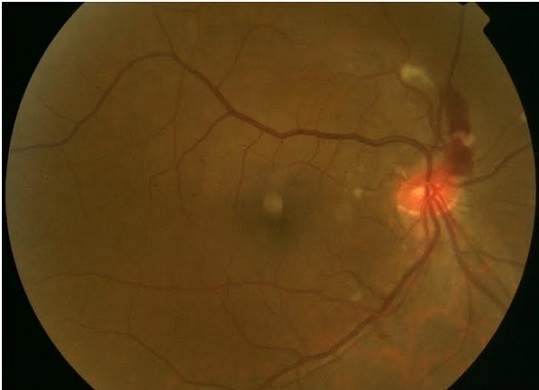
Rétinophotographie: hémorragie papillaire et nodules dysoriques

